# Exploring the Shallow End; Estimating Information Content in Transcriptomics Studies

**DOI:** 10.3389/fpls.2012.00213

**Published:** 2012-09-10

**Authors:** Daniel J. Kliebenstein

**Affiliations:** ^1^Department of Plant Sciences, University of CaliforniaDavis, CA, USA

**Keywords:** transcriptomics, information content, microarray, RNAseq, sequencing depth, factorial genomics, eQTL, genetical genomics

## Abstract

Transcriptomics is a major platform to study organismal biology. The advent of new parallel sequencing technologies has opened up a new avenue of transcriptomics with ever deeper and deeper sequencing to identify and quantify each and every transcript in a sample. However, this may not be the best usage of the parallel sequencing technology for all transcriptomics experiments. I utilized the Shannon Entropy approach to estimate the information contained within a transcriptomics experiment and tested the ability of shallow RNAseq to capture the majority of this information. This analysis showed that it was possible to capture nearly all of the network or genomic information present in a variety of transcriptomics experiments using a subset of the most abundant 5000 transcripts or less within any given sample. Thus, it appears that it should be possible and affordable to conduct large scale factorial analysis with a high degree of replication using parallel sequencing technologies.

## Introduction

The development of methods for directly measuring thousands of transcripts simultaneously, transcriptomics, has been a major factor in the advancement of biological studies and the creation of new fields like genomics and systems biology. The use of transcriptomics has spread to nearly every field of biological study for example, genetics, biochemistry, ecology, and evolution. This has allowed for better understanding of how an organism’s transcriptome is structured by regulatory and evolutionary pressures and more fundamentally allowed the identification of the function for innumerable new genes. Recent technological advancements have led to the rapid conversion from microarray based transcriptomics to RNAseq based transcriptomics largely because of increased breadth of organisms for which RNAseq is possible. RNAseq methodology also provides a new ability to study other aspects of transcriptomics such as splicing and processing.

An emerging area of systems biology that requires full utilization of transcriptomics is the area of factorial biology, i.e., the biological response of multiple treatments or conditions. Modern systems biology and genomics have done a great job of studying individual genetic variants or regulatory networks in isolation but it is rapidly becoming obvious that this provides a limited view of an organism. Instead of using networks in isolation, organisms must integrate the signal inputs from all of these networks to measure and properly orchestrate a phenotype. Unfortunately measuring this integration requires factorial experiments where the organism is manipulated according to at least two separate treatments. For instance, there have been systems genetic studies of how knockouts in all pairwise combinations of *S. cerevisiae* genes combine to affect growth (Segre et al., 2005; Roguev et al., 2008). However, these studies are limited to the ability to robotically control the organism and measure a single phenotype within a 5000 × 5000 gene matrix of pairwise epistatic combinations, in this case a single replicate of the entire genetic matrix would require 25,000,000 genotypes. This factorial nature generates an experiment of the size where complete transcriptomics upon 25,000,000 lines is not considered technically or financially feasible.

Another approach to the same goal of systems genetics has been to utilize crosses between natural genotypes to allow segregation to shuffle 100 to 1000 s of polymorphisms and then measure the transcriptome in the resulting progeny (Brem et al., 2002; Brem and Kruglyak, 2005; Kliebenstein et al., 2006a; West et al., 2007). However, the population sizes barely scratch the possible combinations of alleles because they typically have less than 500 individuals for a population that may have at least 1000 different causal polymorphisms with the ability to affect the transcriptome (Chan et al., 2011). Thus, this example would require a population of 1,000,000 individuals to sample the 1000 × 1000 matrix of all possible pairwise combinations between the causal polymorphisms to fully interrogate the factorial nature of the natural variation network. In this case the 500 original individuals would only sample 0.05% of the potential genetic matrix. Thus, there is a need to develop approaches to allow genomics of much larger genotype collections to fully understand how networks may vary in nature.

Systems regulation is another area with an emerging need for factorial experiments with transcriptomics. A prime example of this is research into the transcriptional circadian clock, which is showing how regulatory networks are central to the function of an organism by integrating numerous inputs (light, heat, metabolism, etc) to properly control the output of the clock (Harmer et al., 2000; Covington and Harmer, 2007; Covington et al., 2008; Harmer, 2009). Thus, a full transcriptomic understanding of the clock in an organism would require a multi-factorial survey of the environment and how variation in all of the external cues combines to shape the organism’s phenotype. Similar observations of massive integration are present in the literature on interactions between biotic and abiotic stimuli and development and the environment suggesting that there may be no isolated regulatory networks further emphasizing the need for massively factorial manipulations involving transcriptomics.

The daunting nature facing the above factorial studies is the vast number of samples that need to be analyzed for transcriptomics. This large sample requirement forces a need (desire?) to develop methods and approaches to quick and cheaply conduct these factorial analyses. One solution may be the use of next generation sequencing technologies that have been shown to have the capacity for high-throughput parallel sequencing of DNA for rapid large scale mapping studies (Tarazona et al., 2011; Monson-Miller et al., 2012). However, the use of next generation sequencing for RNAseq has largely focused on the identification and measuring of more transcripts, i.e., deep sequencing, to capture the expression of all/most genes present in the transcriptome. The results from these experiments have largely already been previously investigated using microarrays with the two approaches leading to the same general observations in *Arabidopsis thaliana* (Kliebenstein et al., 2006b; Van Leeuwen et al., 2007; Zhang et al., 2008; Gan et al., 2011).

One difficulty with transcriptomics optimization is that transcriptomes have significant co-expression that is largely driven by the shape of the underlying regulatory network (Velculescu et al., 1997; Ge et al., 2001; Hirai et al., 2005; Obayashi et al., 2007; Chan et al., 2011). This co-expression structure of the transcriptome has often led to the goal of finding a specific subset of transcripts that measure key nodes of this network and the entire state of the transcriptome could theoretically be described by monitoring the expression of a small set of select genes. However, finding this set has been elusive since the key nodes often change depending upon the biological question. An alternative would be to take a randomized set of genes. In addition, even if a specific subset could be identified this still requires specialized technology to measure these specific genes that typically do not allow for the enhanced throughput required to conduct massive factorial or quantitative genomics experiments (Heinrich et al., 2012). Given the similarity in transcriptomics results between the platforms, I theorized that it may be possible to utilize shallow RNAseq analysis for factorial transcriptomic studies by measuring where the information lies in microarray transcriptome studies. This should help to optimize the approach to factorial analysis with transcriptomics.

One potential solution to this conundrum would be to utilize the parallel sequencing capacity of next generation sequencing technologies to sequence transcriptomes at a shallow depth for the factorial experiments (Kumar et al., 2012; Monson-Miller et al., 2012). This could then be input into a network architecture to analyze the transcriptomic data as if it were physiological measurements (Kliebenstein et al., 2006a; Kliebenstein, 2009b; Kerwin et al., 2011). A frequent retort to this idea is that this approach would be a biased sample towards the most expressed genes which could not possibly provide the information that would be desired about how the transcriptome behaves in factorial experiments. It is true that this would be a biased sample but it is currently not known how much of the total possible information present in a transcriptomic study this sample would actually contain.

In this study, I conduct a quantitative analysis of the information contained in several transcriptomic experiments to test how much transcriptome information can be obtained using shallow sequencing of factorial experiments. To accomplish this, I apply a Shannon Entropy analysis to existing transcriptomic datasets to measure the information content of expression biased subsets in comparison to the total dataset (Shannon, 1948). This is applied to three different potential uses of transcriptomics in factorial studies, a general analysis of the co-expression network in the *Arabidopsis* transcriptome, an expression QTL (eQTL) analysis and finally a temporal analysis of the circadian clock output network. In all instances, the data suggests that it should be possible to obtain at least 80% of the information present in a transcriptomic study by only measuring the top 10% of the transcripts within a sample.

## Materials and Methods

### Shannon entropy information content for transcriptomics

I utilized Shannon Entropy to estimate the information content within a transcriptomics experiment (Shannon, 1948). Shannon Entropy has previously been used to assess the information content in DNA and protein sequence (Schneider, 2000; Weiss et al., 2000; Adami, 2004). Shannon Entropy has also been utilized with transcriptomics to test the informational benefit of co-expression or data reduction analysis (Sangurdekar et al., 2006; Cangelosi and Goriely, 2007). In these approaches, Shannon Entropy was utilized to maximize the information obtained. In this paper, I utilize Shannon Entropy to estimate how much of a final transcriptomic result is contained in specific subset of genes to identify the depth of sequencing required to obtain the majority of transcriptomics information. Shannon Entropy requires the transcripts to be grouped into the information states that they provide. Shannon Entropy was calculated using the equation *H*(*X*) = − ∑*_i_ p_i_*log_2_*p_i_*. where H(X) is the information present in a gene set of a transcriptomics experiment where the transcripts occur in *i* different networks or groupings and *p_i_* is the fraction of the total transcripts within that gene set.

### General scale free network information content

To query the information in a generic microarray experiment, I utilized previously published analysis that grouped the *Arabidopsis* transcriptome into co-regulated modules (Mentzen and Wurtele, 2008). These groupings were generated using a wide range of experiments including development, biotic stress, abiotic stress, etc. and the networks showed a typical scale free topology suggesting that it is a good model for how *Arabidopsis* transcripts may be co-regulated into modules. In this analysis, *p_i_* is the measured probability of obtaining a transcript that measures the expression of network *i* from a specific set of transcripts. Microarray data for this analysis was obtained from a previous analysis of specific insertional mutants in enzyme and regulatory genes and transcripts were ranked on their expression level (Sønderby et al., 2007, 2010; Wentzell et al., 2007; Kerwin et al., 2011). The top transcripts subsets were identified and placed into their respective networks. Shannon Entropy was then calculated as above.

### eQTL information content

For testing the information in an eQTL experiment there were two ways to consider the information gleaned from this experiment. The first is via co-regulation modules which would be similar to the above analysis. The second class of information gleaned from an eQTL experiment is the pattern of QTLs found across the genome. To test the information content in genetic architecture obtained by transcriptomic analysis of a RIL population, I grouped the data by where the eQTLs were identified using a given number of transcripts. Microarray data for this analysis was obtained from the previously published eQTL analysis of the Bay x Sha RIL population (Loudet et al., 2002; Kliebenstein et al., 2006a; West et al., 2007). The transcripts were ranked on their average expression across the RIL population. The top transcripts subsets were identified and the detected eQTL for these transcripts were placed at their respective positions. For this analysis, *p_i_* is the measured probability of obtaining an eQTL at position *i* for a specific set of transcripts. Shannon Entropy was then calculated as above. To alter the resolution of mapping the original 1 cM QTL bins into which all eQTL were placed was changed into 5 and 10 cM bins for the respective model comparison and the Shannon information re-estimated for the same transcript sets (Loudet et al., 2002; Kliebenstein et al., 2006a; West et al., 2007). Shannon Entropy was then recalculated for each different recombination resolution population.

### Temporal clock output information content

To measure the information present in a temporal analysis and its fluctuation over time, I utilized previously published circadian clock microarray data (Covington and Harmer, 2007; Covington et al., 2008). The data was used to estimate information both on a CT group based approach and the scale free network model. For the CT group approach, the transcripts were grouped into 1  and 0.25 h bins as previously described to estimate how temporal resolution alters the information content estimate (Covington et al., 2008; Kerwin et al., 2011). For every time point, the transcripts were ranked on measured expression level and the top transcripts subsets were identified and placed into their respective bins. In this analysis, *p_i_* is the measured probability of obtaining a transcript that measures the expression of circadian bin *i* from a specific set of transcripts. Shannon Entropy was then calculated as above.

Shannon Entropy across the time course was also estimated by utilizing the scale free regulon network for every time point to estimate how this approximation of information content may be conditional. For every time point, the transcripts were ranked on measured expression level and the top transcripts subsets were identified and placed into their respective networks. In this analysis, *p_i_* is the measured probability of obtaining a transcript that measures the expression of network *i* from a specific set of transcripts. Shannon Entropy was then calculated as above.

## Results

### Network co-expression and transcriptome information content

To estimate the information content within a transcriptome study, I utilized the Shannon Entropy measure. Shannon Entropy applies to transcriptomics by suggesting that the information potential of a transcriptome is related to the number of transcripts and their co-expression independence. In this system, the maximally informative transcriptome would have no co-expression between any transcripts (Shannon, 1948). In this situation, all transcripts would be equally and independently informative leading to a Shannon Entropy of 14.5 bits for the 22,746 gene transcriptome that can be measured using the ATH1 affymetrix microarray. However, the genes within the transcriptome are not independently expressed and instead show a scale free network of co-expression relationships (Ma et al., 2007; Mentzen and Wurtele, 2008). Recalculating the Shannon Entropy of the ATH1 transcriptome using the proposed scale free network structure leads to a maximal value of 7.1 bits (Mentzen and Wurtele, 2008). Thus, the co-expression nature of the transcriptome decreases the potential information that is potentially present in a transcriptome by nearly 1/2. However, this raises the possibility that it should be feasible to measure or capture most of the information in a transcriptome utilizing a subset of transcripts rather than a complete sampling of all transcripts.

**Figure 1:**
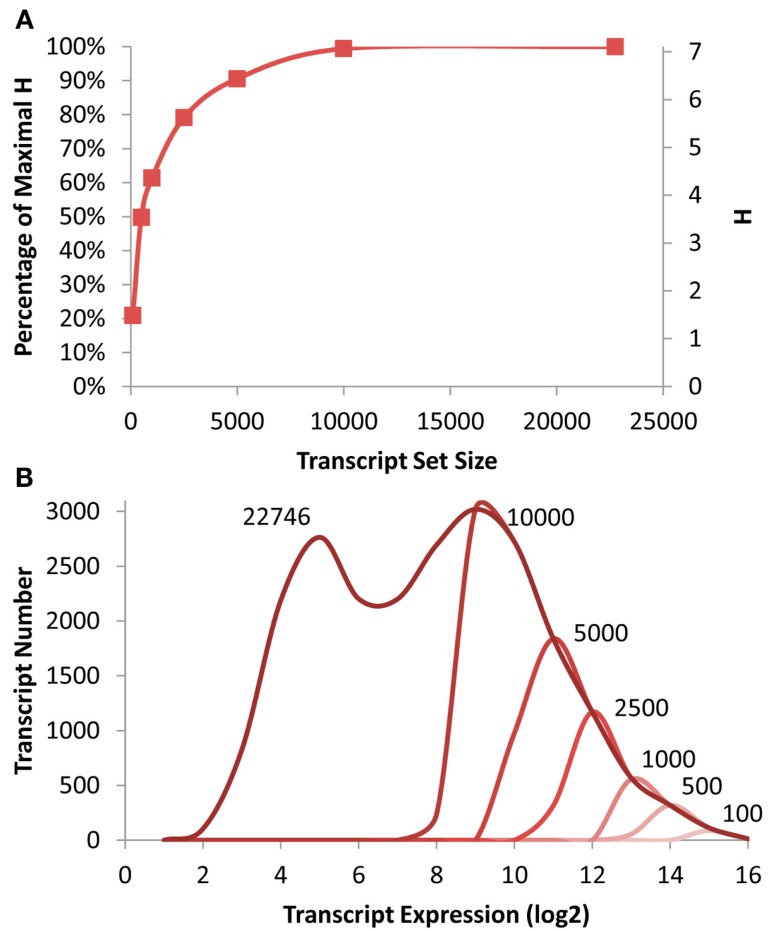
**Transcriptomic information in *Arabidopsis***. **(A)** Shown is the absolute and fractional Shannon Entropy of the *Arabidopsis* transcriptome when limiting the analysis to gene sets of the *X* most expressed transcripts ranging from the 100 top expressed genes to the entire dataset as shown on the *X* axis. **(B)** Shown is the frequency distribution of the average transcript expression level in the microarrays used for this analysis. The darkest line shows the distribution for all transcripts with decreasing red indicating the smaller transcript subsets down to 100 being the lightest red as labeled.

To measure the level of information present in specific gene subsets that would be similarly biased to that expected from a shallow RNAseq analysis, I obtained previously published microarray data investigating the effect of T-DNA mutants in enzymatic and regulatory genes (Sønderby et al., 2007, 2010; Wentzell et al., 2007; Kerwin et al., 2011). The genes were then ranked on their average expression in the dataset and the top 100, 500, 1000, 2500, 5000, 10,000 genes were identified and placed into their proposed scale free network membership within the *Arabidopsis* transcriptome (Mentzen and Wurtele, 2008). The information present in these subsets was recalculated and compared to that present in the full ATH1 array (Figure 1). Interestingly, this suggests that it is possible to approximate 80% of the whole transcriptome level information by only measuring the top 2500 transcripts and that the top 5000 would yield nearly 90% of the information content (Figure 1).

To investigate how much of the distribution of expression levels these subsets cover, I plotted the distribution of expression levels for the whole transcriptome and each of the specific subsets. Interestingly even the top 10,000 transcripts only cover approximately 1/2 of the expression range yet cover nearly 99% of the information content as estimated by the Shannon Entropy. This is possible because each network contains transcripts with a diverse range of expression levels (Hirai et al., 2005, 2007).

### eQTL transcriptome information content

One factorial experiment where transcriptomics is already being conducted is in the analysis of natural variation (Kliebenstein, 2009a). In these studies a segregating population derived from genetically diverse parents is analyzed with transcriptomics to identify the positions in the genome that control natural variation in transcript abundance (Jansen and Nap, 2001; Brem et al., 2002; Schadt et al., 2003; Keurentjes et al., 2007; West et al., 2007). The causal positions of eQTLs are split into either cis- or trans-polymorphisms depending on if the location mapped for causality overlaps with the physical position of the transcript being tested for eQTLs (Hansen et al., 2008; Kliebenstein, 2009a). A researcher can obtain information about the structure of the organism’s co-expression networks as well as information about the genetic position of the causal polymorphisms. The co-expression network architecture information discovered using eQTL data is similar to the above scale free studies and shows similar information content behavior as previously described (Figure 1; Lee et al., 2006; Keurentjes et al., 2007; Jiménez-Gómez et al., 2011). Thus, I focused on the information content present within the genetic position of the causal loci for eQTLs.

**Figure 2:**
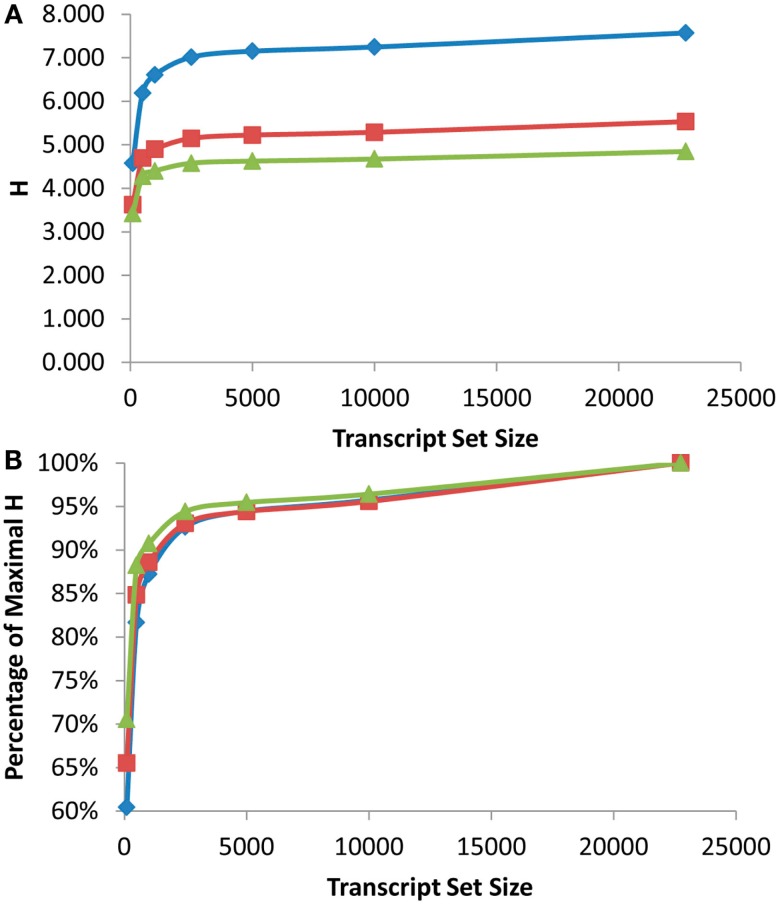
**Shannon Entropy estimation of information in eQTL mapping experiments**. eQTL mapping with different genetic resolution was modeled and the Shannon entropies were estimated using the previously published Bay x Sha RIL dataset. Diamonds show eQTL mapping with 1 cM resolution, squares are 5 cM and triangles are 10 cM. **(A)** Shows the estimated Shannon entropy for the different gene set sizes. **(B)** Shows the fraction of maximal Shannon entropy per gene set size for the same data.

As was found with the scale free network analysis, there was a significant inflection point at which minimal further information was obtained by measuring more transcripts. In the eQTL analysis, this inflection point was reached between the top 1000 and 2500 expressed transcripts across the RILs with the top 2500 expressed transcripts providing 90–95% of the genetic information present in the different recombinant frequency populations (Figure 2). Even measuring the top 100 expressed transcripts obtained between 60 and 70% of the information present (Figure 2B). Thus, conducting a shallow sequence analysis of eQTL studies would only require about 10% of the transcripts to be measured to provide the majority of the genetic information content present in the population.

Another major factor in any future eQTL experiment will be the size of the population utilized for the analysis (Mackay, 2001; Buckler et al., 2009; Kliebenstein, 2009a). A fundamental component of increasing population size is the improved recombination resolution that is provided by additional lines (Mackay, 2001). To test impact upon information obtained by increasing the population size and recombination resolution, I modeled what would occur with changing the resolution of the eQTL mapping from 1 to 5 to 10 cM or the equivalent of a 10-fold range in RIL population sizes. This showed a significant increase in information obtained from an eQTL experiment with elevated recombination resolution with the largest jump being from 5 to 1 cM resolution. Unfortunately the original mapping population does not have additional recombination resolution to test the impact on information content by going beyond 1 cM (Loudet et al., 2002; West et al., 2006). This analysis does allow a more empirical assessment of how to design partition transcriptome sequencing in future eQTL experiments. Future experiments will yield vastly more information by conducting shallow sequencing on massive populations in comparison to deep sequencing on small population sizes. This is precisely illustrated by the fact that measuring the top 500 transcripts in a population with 1 cM resolution provided more information than measuring the all transcripts with only 10 cM resolution (Figure 2A).

**Figure 3:**
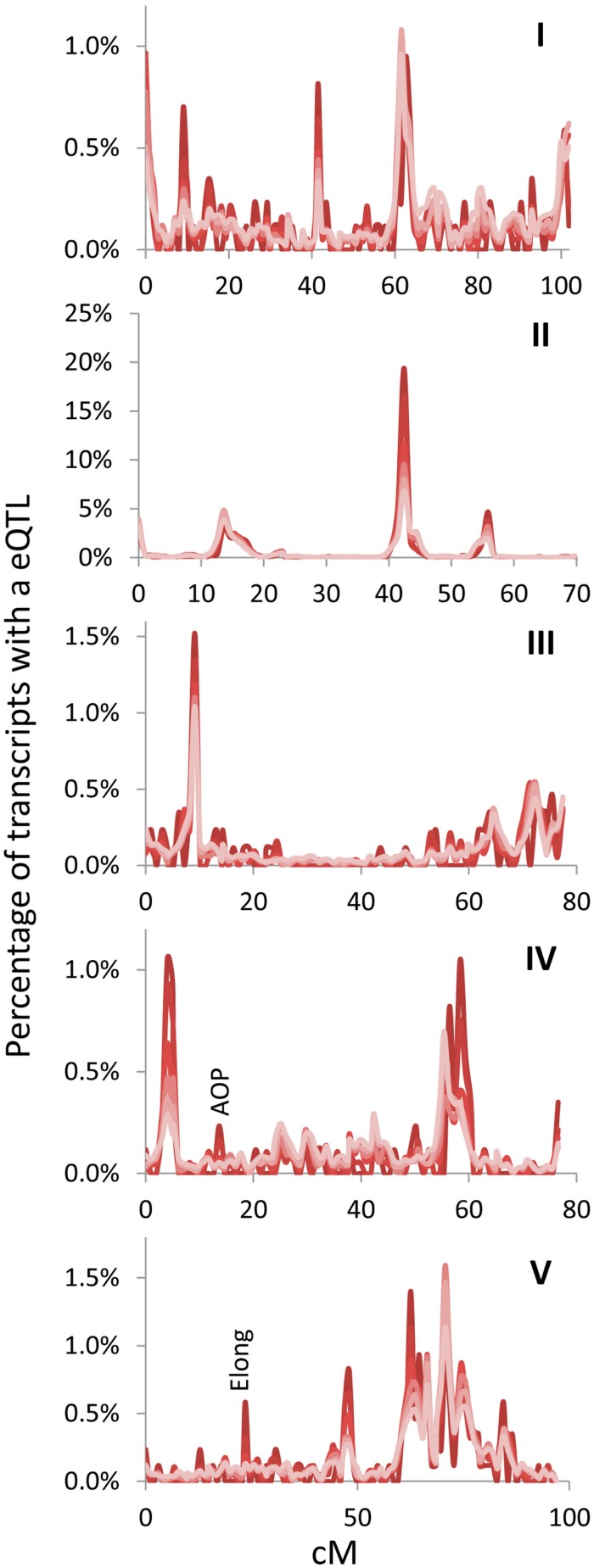
**Hotspot identification with different size gene sets**. eQTL hotspot identification across the five *Arabidopsis* chromosomes are shown with the darkest red being for the top 500 expressed genes and progressively lighter shades of red representing 1000, 2500, 5000, 10,000, and the lightest red being all 22,746 transcripts in the experiment. eQTLs per every cM are plotted without smoothing. AOP and Elong show the position of known pathway specific trans eQTL hotspots for the glucosinolate pathway. Chromosomes are shown in Latin numbering.

The above empirical observations of how to structure a eQTL experiment do run somewhat counter to the common assumption that every transcript must be measured per line because of an inherent desire to not leave any information behind. Thus, I next mapped the position of all eQTLs for the different transcript sets (500, 1000, 2500, 5000, 10,000, and all) to visualize if they were providing similar information content. The 100 transcript set was dropped for visualization because it only provided 60% of the information in a 1 cM population. This analysis showed that the pattern of eQTLs across the genome were nearly identical across the different transcript sets (Figure 3). There were a couple of genomic positions that were more evident in the smaller transcript sets of 500 and 1000 which might be considered as false positives. However, two of these (*AOP* and *Elong*) are network specific hotspots wherein the causal genes are known (Wentzell et al., 2007; Kerwin et al., 2011). This shows that the Shannon Entropy information estimates are accurate with regards to the information content and that it is possible to capture the vast majority of an eQTL experiment’s information using a shallow sequencing approach.

### Circadian network output information content

One complication with directly applying the Shannon Entropy analysis to the information present within transcriptomes is that transcripts will dramatically change their expression level over large temporal, developmental or environmental shifts. This will in turn lead to large changes in relative expression rank that may influence the above estimates. To query if rank changes alter the information content estimates and the potential utilization of shallow sequencing, we utilized a circadian temporal time course (Covington and Harmer, 2007; Covington et al., 2008). In this time course, genes such as those involved in photosynthesis will oscillate in rank from highly expressed in the supposed day to low expression in the night allowing an investigation into how Shannon Entropy information content estimates for transcript subsets may fluctuate under this regime (Harmer, 2009).

**Figure 4:**
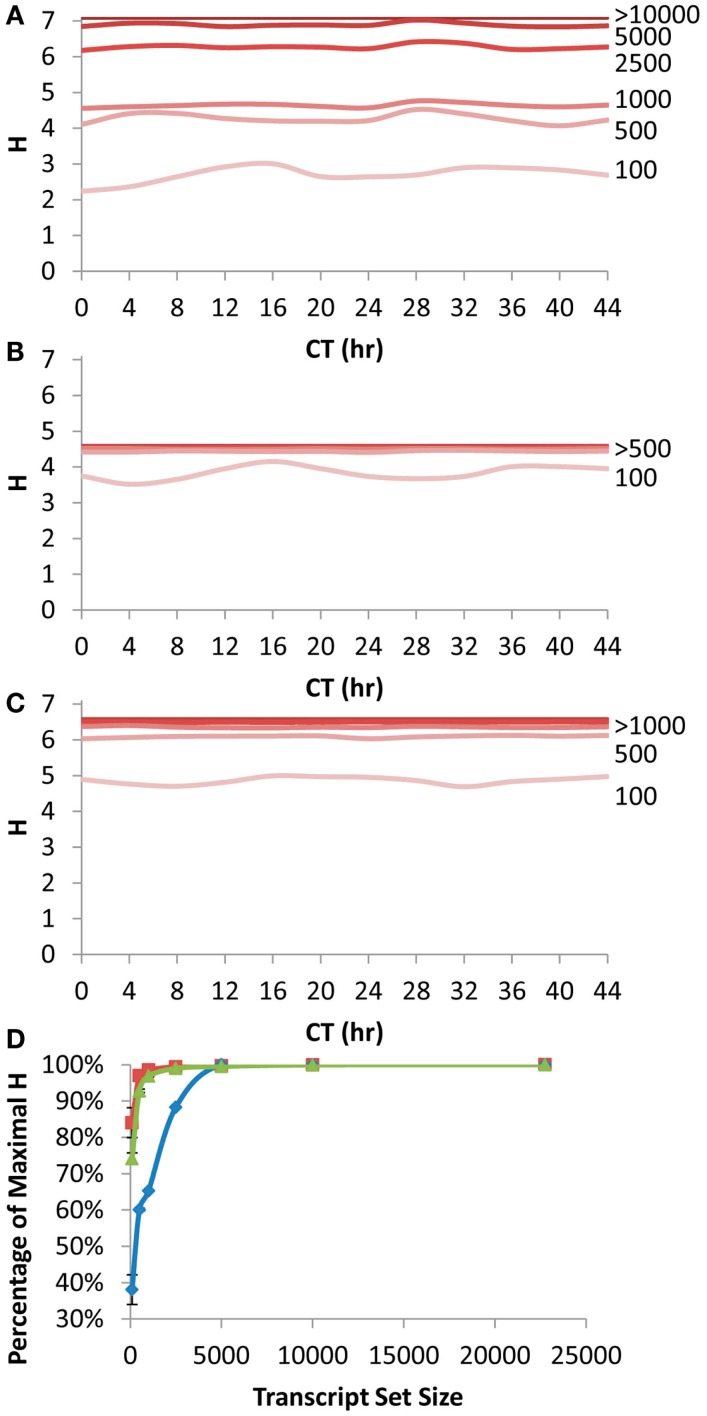
**Shannon Entropy estimation of information in circadian time course experiment**. A previous microarray analysis of a 44 h circadian time course with the Affymetrix ATH1 microarray was obtained to analyze the Shannon Entropy within the analysis. The genes were independently ranked in each time point and the Shannon entropy for the different gene sets analyzed for each time point. **(A)** The estimated Shannon entropy across the different time points (CT hour) using the scale free transcript network. Transcript set sizes are as labeled. The lines for 10,000 and 22,746 transcripts are indistinguishable and labeled >10,000. **(B)** The estimated Shannon entropy across the different time points using the 1 h CT group network assignment from the same data. Transcript set sizes are as labeled. The lines for 500, 1000, 2500, 5000, 10,000, and 22,746 transcripts are indistinguishable and labeled >500. **(C)** The estimated Shannon entropy across the different time points using the 1/4 h CT group network assignment from the same data. Transcript set sizes are as labeled. The lines for 1000, 2500, 5000, 10,000, and 22,746 transcripts are indistinguishable and labeled >1000. **(D)** The average fraction of maximal Shannon entropy per gene set size across all time points. Diamonds show the scale free network, squares show the 1 h CT group network while triangles are the 1/4 h CT group networks. Standard deviation is shown. If bars are not visible then the standard deviation is smaller than the symbol for that point.

We first queried how the rank fluctuations across a circadian time course would alter the estimates of information contained in different transcript subsets for the scale free network (Figure 4A). The Shannon Entropy was estimated independently at each time point using the ranking of transcripts at that time point to select the transcript subsets. The temporal time course had a statistically significant impact on the information content but only when choosing the top 100 or top 500 transcripts per time point (ANOVA, *P* < 0.05; Figure 4A). In contrast, there was no significant impact of the time course upon the estimated Shannon Entropy information when using transcript subsets of 1000 or greater (Figure 4A). As previously noted (Figure 1A) it took only 10–20% of the total transcripts at any given time point to obtain 90% or more of the information contained in the full transcriptome (Figure 4D).

Another way to measure the circadian clock information is to utilize a network list that is derived directly from previous circadian time courses. This is done by establishing at what time in the circadian time course (CT – circadian time) the transcript has its peak expression and grouping together genes with a similar CT peak (Kerwin et al., 2011). We utilized previous CT peak estimates to make two network groupings (CT Groups), one where the transcripts are grouped into 1 CT hour bins and the other in 15 min bins (Covington et al., 2008; Kerwin et al., 2011). The 15 min CT grouping identifying more information than the 1 h CT groups (Figures 4B,C). This is because the 15 min grouping has more resolution than the 1 h grouping leading to the potential for more information to be obtained. In contrast to the scale free network, both the 1 h and 15 min CT groupings were able to capture >90% of the information in the circadian time course with only 500 transcripts (Figure 4D).

**Figure 5:**
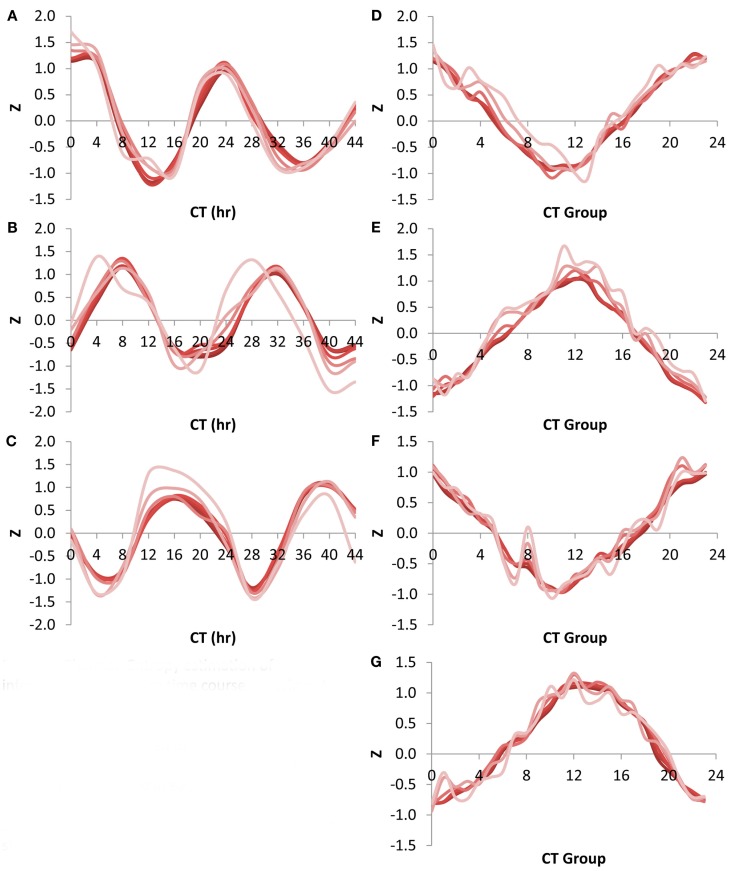
**Shannon Entropy estimation of information in circadian time course experiment**. A previous microarray analysis of a 44 h circadian time course with the Affymetrix ATH1 microarray was obtained to analyze the Shannon Entropy within the analysis. The genes were independently ranked in each time point and the Shannon entropy for the different gene sets analyzed for each time point. In all plots different shades represents the different gene set sizes ranging from 22,746 in the darkest shade to 100 in the lightest shade. **(A)** Expression of CT Group 0 across time. **(B)** Expression of CT Group 8 across time. **(C)** Expression of CT Group 16 across time. **(D)** Expression of the 24 CT Groups at time CT 0. **(E)** Expression of the 24 CT Groups at time CT 12. **(F)** Expression of the 24 CT Groups at time CT 24. **(G)** Expression of the 24 CT Groups at time CT 36.

Using the Shannon Entropy has a long history of accurately estimating the ability to recover information from a subset of a dataset. However, this is an abstract number and to base it in more common visualizations, I investigated the ability of these transcript subsets to recapture the circadian oscillation using the CT group method. This was first done by looking at the network expression of the CT 0, 8, and 16 groups (i.e., the genes that peak at 0, 8, and 16 CT hour) across the circadian time course using the different size transcript subsets. Intriguingly, all transcript subsets involving the top 500 or more transcripts per time point could capture a highly accurate and reproducible image of the transcriptional oscillation (Figures 5A–C). While the 100 top transcripts at any given time could capture the oscillatory behavior for all three CT groups it had difficulty accurately regenerating the transcriptional clock (Figures 5A–C). I also investigated the ability of the different sized transcript subsets to capture the circadian clock at single time points using the average expression across the different CT groups (Kerwin et al., 2011). In agreement with the analysis of individual CT groups across time, this showed that transcripts sets of more than 500 transcripts could accurately measure the oscillatory behavior of the transcriptional clock at any of the time points (Figures 5D–G). Again, the 100 transcript sets while generating the impression of the oscillation could not estimate it as precisely. This agrees with the observation that 500 transcripts can capture most of the Shannon Entropy information using the CT group method to measure the clock.

### Illumina sequencing depth

**Figure 6:**
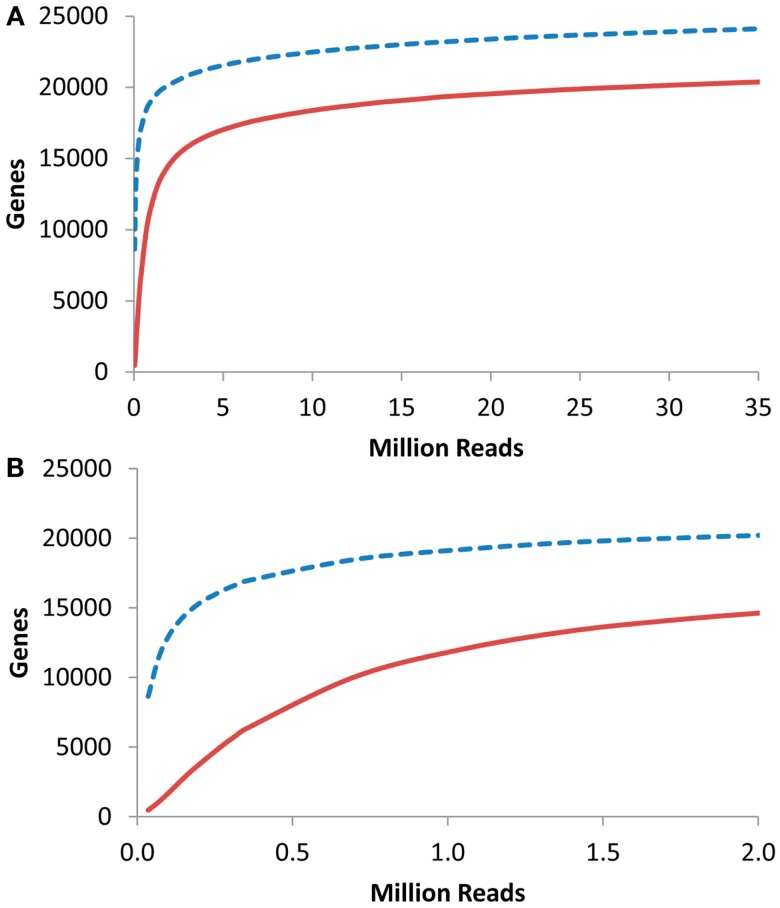
**Gene coverage in *Arabidopsis* RNAseq**. Using a previous analysis of the *Arabidopsis thaliana* transcriptome via RNAseq, I plotted the number of genes identified with 1 (dashed blue line) or 10 (solid red lines) reads per gene in a given number of RNAseq reads. **(A)** Full plot. **(B)**. Close up of plot in **(A)**.

I next proceeded to investigate at what depth an RNAseq based transcriptome approach would have to be conducted to identify the majority of transcriptome information. To accomplish this, I obtained previous aRNAseq experiments that contained the pooled reads from the leaves of seven 3-week-old *Arabidopsis* plants and tested how many genes were identified with 1 or 10× coverage when using a given number of reads (K. Nozue and J. N Maloof, University of California, Davis, personnel communication). This showed that only about 250,000 reads were necessary to obtain 10× coverage on 5000 transcripts within an RNAseq experiment (Figure 6). 5000 transcripts is the level that was able to routinely measure over 90% of the Shannon Entropy information in a transcriptomics study suggesting that it is possible to do shallow RNAseq with only 250,000 reads per sample. Given that most modern technologies are conservatively giving 100 million reads per lane, this would suggest that multiplexing of up to 400 samples per lane would still allow for over 90% of the transcriptomic information content to be obtained in each sample. More importantly, this suggests that it is possible to conduct the high levels of independent biological replicates that are required for precise statistics when using RNAseq or any other technology (Auer and Doerge, 2010, 2011; Auer et al., 2012).

## Discussion

The use of Shannon Entropy to measure the information content that is measurable in microarray analysis of *Arabidopsis* showed that it is possible to capture 90+% of the information present in most microarray studies by measuring only a given set of the top expressed transcripts within a given condition. In all instances measuring the top 5,000 transcripts, approximately 20%, in a given array allowed the ability to obtain 90–99% of the information present in that given microarray. The estimate of information captured in a given experiment was dependent upon the type of information being queried. The use of a scale free transcript co-expression network consistently obtained the highest level of information, but interestingly the maximal information from this network was less than 1/2 of what would have been expected if all transcripts are independent. Thus, while the co-expression of transcripts is problematic for downstream interpretation, it provides the ability to conduct shallow sequencing to obtain most of the information within a given experiment. The analysis on Shannon Entropy information herein presented suggests that it should be eminently possible to use shallow sequencing of between 10 and 20% of all transcripts to capture the 90% or more of information within a given transcriptomic experiment. Using this data reduction approach to capture most of the transcriptomic information in a random transcript subset starts to open up the possibility of conducting transcriptomic analysis on massive factorial experiments by utilizing shallow RNAseq analysis of the transcriptome. A complement to this strategy will require an improvement in our ability to automate RNA isolation and RNAseq libraries as this is the next bottleneck in the process of transcriptomics on factorial experiments but progress is being made (Kumar et al., 2012).

### What is this information

One difficulty with the use of Shannon Entropy to estimate information in a biological experiment is that the Shannon Entropy is an abstract value which doesn’t provide the ability to directly visualize or grasp what is being measured. This abstraction directly raises the question of “what is this information being measured?” A simple way to think about the information content is to equate it to how many pixels in an original image are required for the human brain to recreate the picture. In a biological context this equates to how many transcripts are required to recreate the biological image in question. For example, it only required the top 1000 transcripts to create a nearly identical image of eQTL distribution across a genome and transcriptional circadian oscillations as was observable using the complete transcriptome (Figures 3 and 5). Thus, as with the image description, the information estimated by Shannon Entropy is not the ability to perfectly recreate each and every pixel or transcript in a dataset but instead to obtain the over-riding patterns or more liberally meaning within the data. In biological data, this information can be considered to be the position of eQTLs in a genomic context, the expression of gene networks or possibly even the physiological status of the plant as described by the fluctuations in the transcriptomes co-expression network.

### Information in a transcriptome versus a gene

The proposal that it is possible to capture the vast majority of information within a transcriptome with a limited subset of transcripts only pertains to the overarching patterns within a transcriptome. This proposal does not suggest that it is possible to identify the information present in each and every transcript utilizing a subset of transcripts. This is equivalent to saying that it is possible to shrink a digital image and maintain the vast majority of information but it is not possible to go backwards in this process. Thus, if an experiments true interest is to measure each and every transcript and the focus is on the specific transcripts, then this shallow RNAseq approach will not likely be sufficient. However, it should be noted that the analysis presented on Shannon Entropy information raises the question of when it is necessary to obtain all of the transcriptome from a given sample for deriving biological inference. If it is possible to approximate the transcriptional clock with only 1000 of the highest expressed transcripts as shown, then it could be argued that there would be no reason to do a circadian time course with deep RNAseq and instead it would be more valuable to expand the time course to involve several treatments and query how the treatment space influences the clocks oscillatory behavior with shallow RNAseq. At the very least, the Shannon Entropy analysis suggests that more careful consideration should be applied about where in the transcriptome the true biological question lays when designing an experiment. Is the biological question in the behavior of each and every transcript in which case deep RNAseq is advisable or is the true biological question of interest in the behavior of the transcriptome to factorial perturbation? In the latter case, shallow RNAseq will be the fastest and most efficient method to ask how a transcriptome behaves under factorial perturbation and hence how signals are integrated within the transcriptome.

### Prior information and information content

It is important at this juncture to point out that the Shannon Entropy information analysis presented however relies on the availability of prior information to frame the question. In the eQTL analysis, the prior information is relatively easy to obtain as it is the genotype of the individual lines that allows the genetic mapped to be parsed into specific bins. In the analysis of the circadian time course and other developmental, physiological or environmental responses the prior information requires the ability to place the transcripts into networks (i.e., scale free network model) or bins (i.e., CT Groups) to allow the groups response to be estimated from a subset (Figures 1, 4, and 5). Thus, in any approach that intends to rely upon shallow RNAseq for factorial transcriptomics there is a need to have this prior information in hand to generate either the scale free network model or CT groups. Generating this prior information could either be from relying upon previous microarray or deep RNAseq experiments either focused on a specific physiological process like the circadian clock and CT groups or broadly focused on transcriptome organization like the broad yet shallow sampling across environmental and developmental states that allowed the generation of the scale free network model. An intriguing possibility is the use of gene-sharing networks to move transcriptome organization information from one species to another possibly allowing the generation of crude but useful scale free models in species without a deep transcriptomics data repository (Li et al., 2012).

## Conclusion

The analysis of transcriptome information content using the Shannon Entropy information shows that >90% of the information within a transcriptomic experiment is accessible using just the top 20% of expressed transcripts within a specific sample. This level of coverage is readily achievable using just a fraction of the sequencing power available in a single run of a next generation sequencing platform thus raising the ability of conducting massively parallel shallow sequencing of RNA samples while still collecting nearly all of the possible information in these transcriptomes. If broadly applied, this shallow RNAseq approach would rapidly facilitate the application of transcriptomics approaches to factorial genetics and regulatory studies that had previously been thought to be off limits to transcriptomics because of financial and technical concerns. Shallow RNAseq surveys of factorial experiments could then allow us to study how the genome perceives and integrates information at a level of precision and resolution far greater than is currently available.

## Conflict of Interest Statement

The author declares that the research was conducted in the absence of any commercial or financial relationships that could be construed as a potential conflict of interest.

## Abbreviations

Nomenclature: QTL, a position in the genome where sequence variation between two genotypes leads to a measurable phenotypic difference between individuals with these genotypes; eQTL, a QTL that alters the accumulation of a specific and defined transcript; cis-eQTL, also called a proximal eQTL; the genomic position of the eQTL shows a tight linkage to the physical position of the gene encoding the measured transcript; trans eQTL, also called a distal eQTL; the genomic position of the eQTL is not linked to the physical position of the gene encoding the measured transcript; H, Shannon Entropy is a mathematical approach to quantify the amount of information contained within a dataset and the output is described in the units of bits; CT, Circadian time with the onset of dawn being CT 0 h; CT Group, Networks of genes as defined by their peak of expression during a circadian time course. For example CT Group 0 represents the genes whose maximal expression is between CT 0 and CT 1.
